# Charting the Metabolic Landscape of the Facultative Methylotroph Bacillus methanolicus

**DOI:** 10.1128/mSystems.00745-20

**Published:** 2020-09-22

**Authors:** Baudoin Delépine, Marina Gil López, Marc Carnicer, Cláudia M. Vicente, Volker F. Wendisch, Stéphanie Heux

**Affiliations:** a TBI, CNRS, INRA, INSA, Université de Toulouse, Toulouse, France; b Genetics of Prokaryotes, Faculty of Biology & CeBiTec, Bielefeld University, Bielefeld, Germany; Max Planck Institute for Marine Microbiology

**Keywords:** natural methylotrophy, ^13^C metabolic flux analysis, nonstationary MFA, *Bacillus methanolicus* MGA3, methanol

## Abstract

Bacillus methanolicus MGA3 is a thermotolerant and relatively fast-growing methylotroph able to secrete large quantities of glutamate and lysine. These natural characteristics make B. methanolicus a good candidate to become a new industrial chassis organism, especially in a methanol-based economy. Intriguingly, the only substrates known to support B. methanolicus growth as sole sources of carbon and energy are methanol, mannitol, and, to a lesser extent, glucose and arabitol. Because fluxomics provides the most direct readout of the cellular phenotype, we hypothesized that comparing methylotrophic and nonmethylotrophic metabolic states at the flux level would yield new insights into MGA3 metabolism. In this study, we designed and performed a ^13^C metabolic flux analysis (^13^C-MFA) of the facultative methylotroph B. methanolicus MGA3 growing on methanol, mannitol, and arabitol to compare the associated metabolic states. On methanol, results showed a greater flux in the ribulose monophosphate (RuMP) pathway than in the tricarboxylic acid (TCA) cycle, thus validating previous findings on the methylotrophy of B. methanolicus. New insights related to the utilization of cyclic RuMP versus linear dissimilation pathways and between the RuMP variants were generated. Importantly, we demonstrated that the linear detoxification pathways and the malic enzyme shared with the pentose phosphate pathway have an important role in cofactor regeneration. Finally, we identified, for the first time, the metabolic pathway used to assimilate arabitol. Overall, those data provide a better understanding of this strain under various environmental conditions.

**IMPORTANCE** Methanol is inexpensive, is easy to transport, and can be produced both from renewable and from fossil resources without mobilizing arable lands. As such, it is regarded as a potential carbon source to transition toward a greener industrial chemistry. Metabolic engineering of bacteria and yeast able to efficiently consume methanol is expected to provide cell factories that will transform methanol into higher-value chemicals in the so-called methanol economy. Toward that goal, the study of natural methylotrophs such as Bacillus methanolicus is critical to understand the origin of their efficient methylotrophy. This knowledge will then be leveraged to transform such natural strains into new cell factories or to design methylotrophic capability in other strains already used by the industry.

## Abstract (precis)

Methanol is inexpensive, is easy to transport, and can be produced both from renewable and from fossil resources without mobilizing arable lands. As such, it is regarded as a potential carbon source to transition toward a greener industrial chemistry. Metabolic engineering of bacteria and yeast able to efficiently consume methanol is expected to provide cell factories that will transform methanol into higher-value chemicals in the so-called methanol economy. Toward that goal, the study of natural methylotrophs such as Bacillus methanolicus is critical to understand the origin of their efficient methylotrophy. This knowledge will then be leveraged to transform such natural strains into new cell factories or to design methylotrophic capability in other strains already used by the industry.

## INTRODUCTION

In the last decade, ^13^C metabolic flux analysis (^13^C-MFA) has emerged as an outstanding experimental method to describe the metabolic states of microorganisms. It has successfully been used to identify new pathways (1), investigate responses to environmental changes (2), improve the titer of cell factories (3), screen strains based on their enzymatic capacity (4), and, more generally, to provide a better understanding of the metabolism of microorganisms (5) such as methylotrophs (6–8). Briefly, ^13^C-MFA exploits a metabolic model and ^13^C-isotope patterns measured from key metabolites to estimate reaction rates consistent with the observed labeling patterns (see references 9 and 10 for reviews). Specifically, cells are grown on a ^13^C-labeled carbon source, metabolites of the central metabolism or constituents of the biomass such as proteinogenic amino acids are sampled and quenched, and the labeling patterns of metabolites are monitored by mass spectrometry (MS) and/or nuclear magnetic resonance (NMR). With NMR, carbon isotopomers are determined; hence, positional information is provided. With MS, isotopologues are identified. A metabolic model is then used to fit these measurements to theoretical data that are simulated by optimizing reaction flux values from the metabolic model. Assuming mass balance, if the measurements are coherent and the topology of the metabolic model is correct, the experimental and simulated data will converge, yielding the estimated reaction fluxes. Finally, ^13^C-MFA provides a flux map: a predicted snapshot of the metabolic fluxes through the organism of interest during the experiment, i.e., its metabolic state. ^13^C-MFA flux maps are conceptually similar to those from flux balance analysis (FBA), a purely *in silico* method that, from a metabolic model (typically at the genome scale), computes the optimal reaction flux distribution to maximize an objective defined in terms of metabolite production, often designed to model cell growth. Importantly, ^13^C-MFA flux maps are estimates based on experimental data, whereas FBA flux maps are purely *in silico* predictions, which can be confirmed by gene deletion analysis or ^13^C-MFA, for example.

Bacillus methanolicus MGA3 is a Gram-positive bacterium that was first isolated in the 1990s from freshwater marsh soil samples after an enrichment culture on methanol at 55°C (11). Its ability to grow quickly and to secrete large quantities of glutamate and lysine in methanol at high temperature make it a good candidate for biotech applications. Methanol is indeed viewed as a promising renewable feedstock because of its abundance and low price (12, 13), while high temperature cultures are less prone to contamination and require less cooling when scaled up (14). Furthermore, B. methanolicus is able to grow in seawater, which is also inexpensive and abundant (15). Promising metabolic engineering studies have already established MGA3 as a cell factory for the heterologous production of cadaverine (16) and gamma-aminobutyric acid (GABA) (17). While the lack of genetic tools must have impaired the development of new applications in the past (19), the establishment of gene expression tools based on theta and rolling-circle replicating plasmids has made B. methanolicus amenable to the overproduction of amino acids and their derivatives (18), and there is hope that recent breakthroughs from CRISPR interference (CRISPRi) will stimulate new innovations (20).

As a facultative methylotroph, B. methanolicus MGA3 can also grow on nonmethylotrophic substrates such as d-mannitol, d-glucose, and d-arabitol. The metabolic pathways involved in the uptake of mannitol and glucose have been described (21) as has the organization of genes involved in mannitol utilization (18). Both substrates enter the cells via a phosphotransferase system (PTS) as mannitol 1-phosphate and glucose 6-phosphate, respectively, and are further converted to fructose 6-phosphate. Arabitol was recently characterized as a fourth source of carbon and energy for B. methanolicus (22). The operon responsible for arabitol assimilation was identified as harboring a PTS system (AtlABC) and two putative arabitol phosphate dehydrogenases (AtlD and AtlF), whose activities were demonstrated in crude extracts. However, as the pathway was not completely characterized, it is unclear whether arabitol is assimilated through arabitol 1-phosphate to xylulose 5-phosphate (Xyl5P) or to ribulose 5-phosphate (Ribu5P) through arabitol 5-phosphate. It has been suggested that both routes operate in parallel in Enterococcus avium and other Gram-positive bacteria (23). A series of omics studies comparing these carbon sources with methanol have contributed to a better understanding of B. methanolicus metabolism at the genome (21, 24), transcriptome (21, 22, 25), proteome (26), and metabolome (27, 28) levels. However, a flux-level description, which could validate previous findings and provide new insights into the facultative methylotrophy of B. methanolicus and its associated metabolic states, is still lacking.

In this study, we designed and performed ^13^C-MFA of the facultative methylotroph Bacillus methanolicus MGA3 growing on methanol, mannitol, and arabitol. Methanol (CH_4_O) and mannitol (C_6_H_14_O_6_) are the best-known carbon sources for this strain (11, 14), with comparable growth rates, while growth on arabitol (C_5_H_12_O_5_) is significantly slower (22) and its assimilation pathway has not yet been fully described. All three carbon sources are probably present in MGA3’s natural habitats, on plant leaves (29) or as plant degradation products (30). With their wide availability and fast associated growth rate, methanol and mannitol are promising feedstocks for industrial applications, while arabitol growth allows the facultative methylotrophy of MGA3 to be studied with a less efficient C source and to finish characterizing its assimilation pathway.

## RESULTS AND DISCUSSION

### *In vitro* assessment of arabitol assimilation.

**FIG 1:**
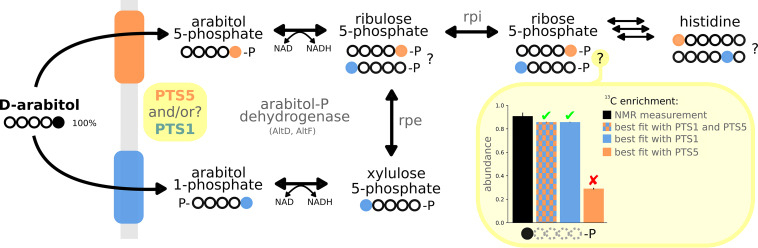
Alternative assimilation pathways of d-arabitol with an example of the measured and expected labeling of Rib5P. Arabitol entry point into the metabolism is expected to be Ribu5P or Xyl5P, depending on the substrate specificity of the PTS system and arabitol phosphate dehydrogenase. Taking [5-^13^C]arabitol as an example, we show that the labeling of downstream metabolites can be used to identify which pathway is operating *in vivo* (Ribu5P, orange; Xyl5P, blue). We exemplify our approach with one data point: the NMR-specific enrichment of Rib5P and the associated estimates from the best fit of different scenarios in which PTS1 or PTS5 are allowed to carry flux in the model (bar plot). Unlike the others, the model in which only PTS5 was allowed to carry flux did not fit the data (red cross mark [✘]). Circles are carbon atoms: solid for ^13^C (●), empty for ^12^C (○), dotted when irrelevant.

The operon responsible for arabitol assimilation in B. methanolicus consists of a PTS system (AtlABC) and two putative arabitol phosphate dehydrogenases (AtlD and AtlF) (22), which are chromosomally encoded and belong to the diverse superfamily of medium-chain dehydrogenases/reductases (MDRs). However, the physiological roles of AtlD and AtlF have not been described to date. Members of the MDR superfamily have high sequence conservation, but sequence-based prediction of their substrate scope is difficult, since wide substrate specificity is common (31, 32) (see Text S1 in the supplemental material for a discussion of their phylogeny). The substrate selectivity of AtlD and AtlF was therefore studied to identify whether arabitol is assimilated via arabitol 1-phosphate dehydrogenase to Xyl5P and/or to Ribu5P via arabitol 5-phosphate dehydrogenase (23) (Fig. 1). The enzymes were purified as N-terminally His-tagged proteins from recombinant Escherichia coli by nickel chelate chromatography. Arabitol phosphate oxidation could not be assayed, because there are no commercial arabitol 1-phosphate or arabitol 5-phosphate standards. Therefore, the reverse reaction was tested, as described in Materials and Methods, with either Xyl5P or Ribu5P as the substrate for NAD(P)H-dependent reduction. A number of potential sugars (d-ribose, d-fructose, d-xylose, d-mannose, l-arabinose, d-arabinose, l-sorbose, d-galactose, d-glucose, ribose 5-phosphate, glucose 6-phosphate, glucose 1-phosphate, fructose 6-phosphate, and fructose 1-phosphate) and sugar alcohols (d-arabitol, d-mannitol, d-galactitol, d-sorbitol, l-arabitol, d-maltitol, d-xylitol, ribitol, and meso-erythritol) were also tested as the substrates, but no significant activity (i.e., >0.05 U mg^−1^) was detected. The kinetic parameters measured for AtlD and AtlF reduction using either Xyl5P or Ribu5P as the substrate are summarized in Table 1. The *K_m_* of 0.07 ± 0.03 mM for AtlD with Xyl5P as the substrate and NADH as cofactor was 2.5 times lower than the value obtained for AtlF (0.18 ± 0.05 mM). With Ribu5P as the substrate, the *K_m_* for AtlD was 17 times lower (1.21 ± 0.42 mM) (Table 1), and for AtlF, no activity was detected. The *V*_max_ for AtlD with Xyl5P as the substrate and NADH as cofactor was 2.7 times higher than with Ribu5P as the substrate (0.49 ± 0.06 U mg^−1^) and 12 times higher than for AtlF with Xyl5P (0.11 ± 0.01 U mg^−1^) (Table 1). NADH was the preferred cofactor over NADPH, with a 10 times lower *K_m_* (0.01 ± 0.01 versus 0.11 ± 0.09 mM). The affinity and NADPH-dependent activity (0.24 ± 0.06 U mg^−1^) could only be determined with AtlD and Xyl5P, as no significant activity was detected for any of the other reactions. Liquid chromatography-mass spectrometry (LC-MS) analyses were performed to confirm the formation of arabitol phosphate in the enzyme reactions catalyzed by AtlD. Although arabitol 1-phosphate and arabitol 5-phosphate could not be distinguished, arabitol phosphate was clearly produced with both Xyl5P and Ribu5P as the substrates (see Fig. S2).

**TABLE 1:** Kinetics data of purified AtlD and AtlF

Condition^*a*^ (protein, substrate, cofactor^*b*^)	Substrate *K_m_* (mM)	*V*_max_ (U mg^−1^)^*c*^	*V*_max_/*K_m_*
AtlD, Xyl5P, NADH	0.07 ± 0.03	1.33 ± 0.23	19
AtlD, Ribu5P, NADH	1.21 ± 0.42	0.49 ± 0.06	0.4
AtlF, Xyl5P, NADH	0.18 ± 0.05	0.11 ± 0.01	0.6

a The following reactions were also analyzed, but no significant activity (i.e., <0.05 U mg^−1^) was detected: AtlD, Ribu5P, NADPH; AtlF, Xyl5P, NADPH; AtlF, Ribu5P, NADH; and AtlF, Ribu5P, NADPH.

b Cofactor *K_m_* values were analyzed using 0.2 mM Xyl5P and were 0.01 ± 0.01 and 0.11 ± 0.09 for NADH and NADPH, respectively.

c The *V*_max_ for AtlD, Xyl5P, NADPH was calculated using 0.2 mM of substrate and 0.3 mM of cofactor, and resulted in 0.24 ± 0.06 U mg^−1^.

10.1128/mSystems.00745-20.5 TEXT S1 Phylogeny of AtlD and AtlF. Download Text S1, PDF file, 0.5 MB. Copyright © 2020 Delépine et al. 2020 Delépine et al. This content is distributed under the terms of the Creative Commons Attribution 4.0 International license.

Overall, these data suggest that AtlD has a major role in arabitol catabolism *in vitro* and that AtlD and AtlF both prefer Xyl5P. This suggests that arabitol is mainly assimilated through the arabitol 1-phosphate pathway. However, assimilation via arabitol 5-phosphate cannot be excluded, since AtlD can also use Ribu5P, albeit with reduced efficiency, as shown both by the kinetic parameters (Table 1) and by the significant residual Ribu5P detected in the enzyme reactions (Fig. S2). Moreover, *in vitro* enzymatic analyses of purified arabitol phosphate dehydrogenase from *E. avium* (23) and Bacillus halodurans (33) showed that both could convert arabitol 1-phosphate and arabitol 5-phosphate into both Xyl5P and Ribu5P. By assessing metabolic operations *in vivo*, ^13^C-MFA experiments should identify which assimilation pathway is actually used *in vivo*.

### ^13^C-MFA experimental design.

Experimental design is a key step to define the isotopic composition of the label input and the isotopic data to be measured, thereby improving both the number of fluxes that can be estimated from a set of isotopic data and the precision of the flux values. To address this point, we used a dedicated program, IsoDesign (34), using as input metabolic models of each carbon source and several labels as described in Materials and Methods. For arabitol and mannitol, good flux precision was obtained with mass spectrometry labeling data from proteinogenic amino acids. This is advantageous for two reasons. First, the experimental setup is simpler than for intracellular metabolites, because no quenching is required and the cellular pellet can just be collected by centrifugation. Second, the labeling can be measured by NMR and MS, providing crucial positional information to distinguish between the two pathways (i.e., via arabitol 5-phosphate or via arabitol 1-phosphate dehydrogenase). Among the different label inputs tested, 100% [5-^13^C]arabitol appeared ideal to identify whether arabitol is converted into arabitol 1-phosphate only or into arabitol 5-phosphate only. In addition, a 9:1 mix of [1-^13^C]arabitol and [2-^13^C]arabitol was used to see if both pathways are active. For mannitol, the best label input was 100% [1-^13^C]. Finally, since methanol is a C_1_ compound, a stationary ^13^C-MFA approach is not suitable, since the amino acids become fully labeled in the isotopic steady state. Nonstationary ^13^C fluxomics should be used instead to follow the incorporation of the tracer after a pulse of labeled substrate; however, significantly more ^13^C data are required for this approach (35, 36) (see Data Set S1).

10.1128/mSystems.00745-20.4 DATA SET S1 Growth curves, processed MS and NMR data, a summary of the reactions modeled, and the relative fluxes in C-mmol/C-mmol of substrate. Raw models and input and output files for influx software and can be downloaded from https://fairdomhub.org/data_files/3269?version=2. Download Data Set S1, XLSX file, 1.9 MB. Copyright © 2020 Delépine et al. 2020 Delépine et al. This content is distributed under the terms of the Creative Commons Attribution 4.0 International license.

All labeling samples were collected in the exponential growth phase at metabolic steady state and analyzed by MS or one-dimensional (1D) ^1^H NMR. The labeling profiles of the analyzed metabolites, as measured by their carbon isotopologue distributions (CIDs), were inspected manually and then used with additional NMR and physiological data to fit a model of B. methanolicus’s central metabolism (Data Set S1).

Some isotopologues of 6-phosphogluconate (Gnt6P) (M + 2 and M + 3) could not be quantified, and the flux through the oxidative pentose phosphate pathway (PPP) could not be accurately resolved. Hence, to exploit these partial data and estimate the flux through the oxidative PPP, we applied the ScalaFlux approach (37). Using such an approach, we obtain a flux through the oxidative PPP (i.e., glucose 6-phosphate dehydrogenase [zwf]) of 0.5909 ± 0.0578 and 0.6919 ± 0.0959 μmol/g (dry cell weight)/s for the two replicates. Next, those data were used to constraint the flux through zwf (as a “measured flux”) through the influx_si optimization process.

### Measurement of physiological parameters.

Assessment of physiological parameters is a prerequisite for flux calculation. Here, B. methanolicus was grown in batches on three different carbon sources. For each cultivation, the growth, consumption, and production rates were determined. Results are given in Table 2. No significant differences between the physiological parameters obtained for the culture replicates were observed. On methanol (batch cultures at 50°C), around 35% of the carbon source was directly evaporated, and biomass and carbon dioxide were the only products formed in detectable amounts. The maximal growth rate obtained here with methanol (0.46 h^−1^) is slightly higher than reported in a previous proteomic study of B. methanolicus MGA3 (0.40 h^−1^) (14) or for the growth of the related strain B. methanolicus PB1 (0.32 h^−1^) (15). Biomass yields did not differ significantly from 0.5 g/g, indicating that approximately one-half of the consumed methanol went to biomass and the other half was oxidized to CO_2_. The growth rates with mannitol and arabitol are consistent with published values (22).

**TABLE 2:** Physiological parameters of B. methanolicus cultures used for metabolic flux analysis^*a*^

Method	Methanol (CH_3_OH) [1-^13^C] MFA bioreactor (*n* = 2)	Mannitol (C_6_H_14_O_6_) [1-^13^C] MFA flask (*n* = 3)	Arabitol (C_5_H_12_O_5_)	
			[1-^13^C] and [2-^13^C] MFA, flask (*n* = 3)	[5-^13^C] MFA, flask (*n* = 3)
Growth rate (h^−1^)^*b*^	0.46 ± 0.002	0.36 ± 0.02	0.15 ± 0.004	0.14 ± 0.02
Biomass yield (g [dry weight]/g substrate)^*b*^	0.55 ± 0.01	0.34 ± 0.08	0.19 ± 0.02	0.23 ± 0.02
Substrate uptake rate (C-mmol/g [dry weight]/h)	30.6 ± 0.6	36 ± 7.3	21.4 ± 1.2	24.5 ± 0.75
CO_2_ evolution rate^*c*^ (mmol/g [dry weight]/h)	7.6 ± 0.04	NA^*d*^	NA	NA
Acetate production rate (mmol/g [dry weight]/h)	ND^*e*^	1.75 ± 0.32	ND	ND

a All cultures were aerobic. The uncertainties shown are standard errors between biological replicates.

b Growth rate and biomass quantities were deduced from OD_600_ measurements in the exponential growth phase.

c Carbon source evolution rates were measured from supernatant samples and analyzed by NMR. Labeled CO_2_ was monitored by MS in the bioreactor’s outgoing gas flow.

d NA, not measured.

e ND, not detected.

B. methanolicus MGA3 is known to overproduce glutamate at up to 50 g/liter (21) on methanol under magnesium or methanol limitation (38, 39). When grown on arabitol or methanol, no metabolite accumulated in the supernatant at concentrations above the NMR detection limit (approximately 100 μM). There was, therefore, little or no metabolite secretion by MGA3 under our chosen methanol and arabitol growth conditions, which is consistent with previous studies and the growth conditions studied here (27, 28). However, acetate was produced at up to 2 mmol/g (dry cell weight)/h in mannitol cultures (yield, 0.3 mol/mol). To the best of our knowledge, acetate production has never previously been reported for B. methanolicus. Based on genomics data (21), we hypothesized that acetate is synthesized from acetyl coenzyme A (acetyl-CoA) in a classical two-step process involving phosphate acetyltransferase (EC 2.3.1.8, BMMGA3_RS15725) and acetate kinase (EC 2.7.2.1, BMMGA3_RS12735). Acetate has, moreover, been discussed at length in the context of overflow metabolism, a special metabolic state in which fermentation pathways are used even though further oxidation (respiration) would be more ATP efficient (40–43).

Overall, we show the reproducible growth characteristics of our cultures and show an unexpected production of acetate that may have an impact for industrial applications, as overflow metabolism leads to carbon and energy waste.

### *In vivo* characterization of arabitol assimilation.

**FIG 2:**
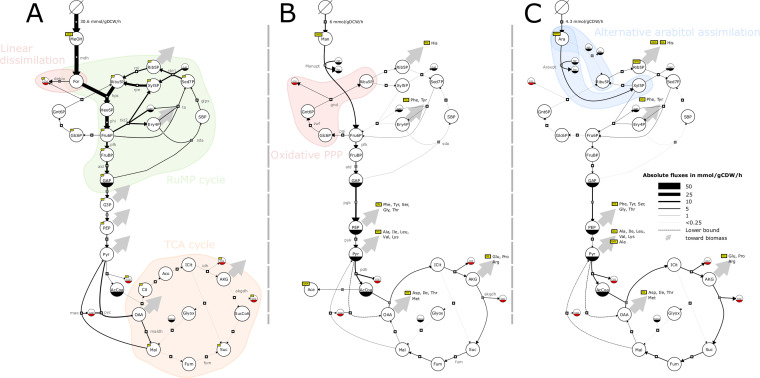
Flux maps of B. methanolicus central metabolism relative to substrate intake for methanol (A), mannitol (B), and arabitol (C). The thicknesses of the reaction lines represent the absolute flux values in millimoles per gram dry cell weight [DCW] per hour. Flux proportions were averaged among the biological replicates (*n* =2 for methanol, 3 for arabitol and mannitol). The direction of the reaction arrows represents the estimated net flux directionality. Dotted arrows represent fluxes obtained by the flux‐minimization method. Large gray arrows represent a flux directed toward amino acid synthesis and biomass requirements for growth. Pathways discussed in the text are represented by a background patch of color. Metabolites are represented by circles and have a solid bottom half if they are duplicated in the same panel, as recommended by the Systems Biology Graphical Notation (SBGN) standard. Metabolites subjected to experimental measurement of ^13^C labeling are marked by yellow boxes. Some intermediate metabolites are not needed for ^13^C-MFA and are consequently lumped in mannitol and arabitol models (B and C); the same metabolites can be explicitly modeled in the methanol model (A), since nonstationary ^13^C-MFA requires all intermediate pools (e.g., G3P, Cit, and Aco). Reactions toward amino acid pools and biomass are not represented.

The *in vitro* enzymatic analyses of the two dehydrogenases, AtlD and AtlF, suggest that both assimilation pathways (i.e., via arabitol 1-phosphate and via arabitol 5-phosphate) may operate *in vivo*. To confirm whether either or both arabitol assimilation pathways are operative in B. methanolicus, as suggested for *E. avium* (23) and Bacillus halodurans (33), we carried out a ^13^C-MFA specifically designed to discriminate between the two pathways (see “*In vitro* assessment of arabitol assimilation”) (Fig. 1). Interestingly, while the possibility to assimilate arabitol through Xyl5P or Ribu5P or both was a free parameter, the optimal solution found during the fitting process exclusively used the route through Xyl5P (Fig. 2C). This indicates that the (low) activity observed *in vitro* through Ribu5P was not present at a detectable level in our cultures and that the PTS system imports arabitol as arabitol 1-phosphate. The kinetic parameters obtained for AtlD and AtlF are in line with the flux data, i.e., entry of arabitol into the pentose phosphate pathway (PPP) via PTS-mediated uptake and phosphorylation to arabitol 1-phosphate followed by oxidation to Xyl5P with AtlD as the major dehydrogenase (Fig. 1 and Table 1).

Overall, these data demonstrate, for the first time, how arabitol is assimilated in B. methanolicus and rule out the hypothesis of additional catabolism through arabitol 5-phosphate and Ribu5P derived from our enzymatic analyses (Table 1) and previous reports in *E. avium* (23) and *B. halodurans* (33).

### *In vivo* operation of the pentose phosphate pathway with the different carbon sources.

B. methanolicus assimilates methanol through the ribulose monophosphate (RuMP) cycle that condenses formaldehyde (For) and ribulose 5-P (Ribu5P) into hexulose 6-P (Hex6P) (44). The regenerative part of the RuMP cycle that maintains a pool of Ribu5P overlaps with the nonoxidative pentose phosphate pathway (PPP). Strong flux through the nonoxidative part of the PPP is therefore expected on methanol, and indeed, a major part of the fructose 6-P (F6P) was entering the nonoxidative part of the PPP (in total, 16.5 mmol/g [dry cell weight]/h, i.e., 61% of the methanol uptake rate) (Fig. 2A). Interestingly, Ribu5P was also recycled through the nonoxidative part of the PPP. A flux of 2.4 mmol/g (dry cell weight)/h (i.e., 9% of the of the methanol uptake rate) through the glucose-6-phosphate dehydrogenase was observed (zwf) (Fig. 2A). The utilization of this route—called the cyclic dissimilatory RuMP pathway (21, 45)—is of particular interest because, intuitively, one imagines that decarboxylation should be avoided when growth occurs on a C_1_ to avoid wasting carbon. This intuition proved to be correct in Bennett et al.’s (46) study of an E. coli synthetic methylotroph in which they knocked out *pgi* to increase the regeneration of Ribu5P through the nonoxidative part of the PPP. However, we cannot exclude the possibility that the oxidative part of the PPP serves as a backup formaldehyde dissimilation pathway when the linear dissimilation pathways become saturated at high methanol concentrations. B. methanolicus is indeed quite sensitive to variations in methanol concentration (47), and we can assume that this critical biological function is tightly controlled. In addition, this route provides NADPH, which is essential for biosynthesis reactions. Overall, those data showed that two-thirds of the methanol was used to regenerate the acceptor molecule for formaldehyde fixation, which is consistent with what has been previously described (48).

Interestingly, mannitol and arabitol are closely connected to the PPP, since mannitol is converted to F6P (just like methanol), whereas arabitol is converted to Xyl5P as discussed above. However, on those carbon sources, the nonoxidative PPP was operating in the opposite direction than when on methanol (Fig. 2B), which is consistent with the absence of necessity to recycle a C_1_ acceptor under those conditions. As expected, the nonoxidative PPP was more oriented toward carbon assimilation on arabitol than on mannitol. On arabitol, the ribulose-phosphate 3-epimerase’s flux (rpe) was in the opposite direction, and the ribose-5-phosphate isomerase’s absolute flux (rpi) flux was almost 2 times higher (Fig. 2B and C) in spite of similar expression levels (22). Simulations carried without transaldolase activity indicated that its activity was essential to fit the labeling data given the network topology used (notably glpx been irreversible) on arabitol. A possibility for future studies would be to take advantage of adaptive laboratory-driven evolution, or overexpression of key enzymes such as transaldolase, to investigate if the PPP could be adjusted to increase growth rates when arabitol is the sole source of carbon and energy. The labeling data suggest that the activity of the oxidative part of the PPP was slightly higher on methanol than on mannitol and almost 5 times lower on arabitol (the estimated absolute values of flux through glucose 6-phosphate dehydrogenase [zwf] were 2.3, 1.8, and 0.5 mmol/g [dry cell weight]/h, respectively) (Fig. 2). However, because the overall rates of the metabolism and the carbon sources differ widely, as can be seen from the substrate uptake rates (i.e., from 30.6 to 4.3 mmol/g [dry cell weight]/h) (Table 2) and the number of carbon atoms (i.e., from 1 to 6), to enable a direct comparison, the absolute fluxes have to be normalized by those values (see Materials and Methods for details). By doing so, the same conclusion was obtained (the estimated zwf flux was 0.45, 0.30, and 0.14 C-mmol/C-mmol of methanol, mannitol, and arabitol, respectively). This is in contrast with the results of previous studies showing that the oxidative part of the PPP is slightly upregulated on mannitol compared to that on methanol (14).

**FIG 3:**
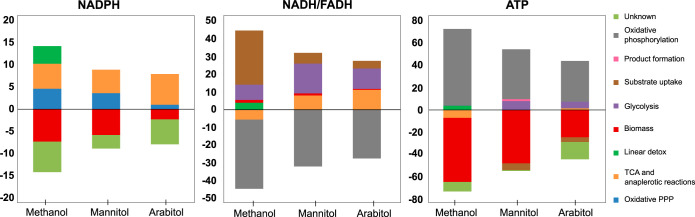
Estimated absolute rates of production and consumption of NADPH, NADH/reduced flavin adenine dinucleotide (FADH_2_), and ATP. Rates (millimoles per gram dry cell weight [DCW] per hour) were calculated as the sum of the estimated flux of the reactions modeled in the MFA that are expected to produce (positive value) or consume (negative value) those cofactors. Values are averages from the biological replicates (*n* = 2 for methanol, 3 for arabitol and mannitol). The growth requirements (“biomass” [red]) were computed from measures on B. subtilis (millimoles per gram dry cell weight) and the growth rates we observed. Production and consumption rates should be balanced under our conditions, and so we marked putative production/consumption rates as “unknown” to complete the balance when needed. The full production of NADH and FADH_2_ is assumed to be consumed in the respiratory chain (“oxidative phosphorylation”) and to produce ATP (with P/O_NADH_ = 1.5, P/O_FADH2_ = 1). We modeled the PTS systems of mannitol and arabitol as consumers of one equivalent ATP (in “uptake”). NADPH production: glucose 6-phosphate dehydrogenase (zwf), phosphogluconate dehydrogenase (gnd), isocitrate dehydrogenase (idh), malic enzyme (mae) and methylenetetrahydrofolate dehydrogenase from the linear detoxification pathway (detox). NADH production: glyceraldehyde-3-phosphate dehydrogenase (pgk), pyruvate dehydrogenase (pdh), 2-oxoglutarate dehydrogenase (akgdh), malate dehydrogenase (maldh), methanol, mannitol, and arabitol dehydrogenase (mdh, manupt, and araupt), and formate dehydrogenase from the linear detoxification pathway (detox). FADH_2_ production: succinate dehydrogenase (fum). ATP consumption: 6-phosphofructokinase (pfk), PTS systems of mannitol and arabitol (manupt and araupt), pyruvate carboxylase (pyc). ATP production: phosphoglycerate kinase (pgk), pyruvate kinase (pyk), succinyl-CoA synthetase (assimilated to akgdh), acetate kinase (out_Ac), and formate-tetrahydrofolate ligase from the linear detoxification pathway (detox).

The RuMP cycle has several variants, which differ in their efficiency (48). Genes for two of these have been identified in MGA3, namely, those encoding the fructose bisphosphate aldolase/sedoheptulose bisphosphatase (SBPase) cycle (50, 51) and the fructose bisphosphate aldolase/transaldolase (TA) cycle (52), which as their names suggest, favor the regeneration of Ribu5P through sedoheptulose bisphosphatase and transaldolase, respectively. It is generally accepted that MGA3 uses the SBPase variant (44). The main evidence for this is the presence of a copy of a characteristic gene of the SBPase variant (*glpX*^P^) on the pBM19 plasmid, whereas there is only one transaldolase gene (*ta*^C^) in the chromosome. Proteomic (26) and transcriptomic (21) studies have also associated *glpX*^P^ with a significant increase in expression in methanol compared with that in mannitol, whereas the expression associated with *ta*^C^ remained constant. According to the ^13^C-MFA, both *glpx* (associated with *glpX*^P^) and *ta* (associated with *ta*^C^) carried a comparable flux; thus, both variants may be active (Fig. 3). This arrangement may serve as a fail-safe to guarantee the replenishment of Ribu5P. Alternatively, since transaldolase activity is essential to fit the isotopic data for growth on arabitol, an advantage of the TA cycle may be that it increases the flexibility of the PPP and allows the regeneration of important precursors such as Ribu5P from different carbon sources.

As expected, the ^13^C-MFA shows that the PPP is critical on methanol, as it overlaps with the RuMP responsible for methanol assimilation. The SBPase variant of the RuMP is active; however, we could not rule out a parallel operation of the TA variant. We suggest that a parallel operation on mixed carbon sources may benefit B. methanolicus to replenish important precursor’s pools.

### *In vivo* operation of the TCA cycle with the different carbon sources.

B. methanolicus has a full gene set for a functional tricarboxylic acid (TCA) cycle and glyoxylate shunt (14, 21). This seems to contrast with some methylotrophs, including some that use the RuMP pathway, that do not need a complete TCA to fulfill their energy requirements (53). Results (Fig. 2A) indicated that the TCA cycle was used much less than the RuMP pathway on methanol, with absolute flux of 0.62 mmol/g (dry cell weight)/h (i.e., 0.12 C-mmol/C-mmol) up to isocitrate (ICit) and almost no flux afterwards (Fig. 2A). This small flux is needed to support the synthesis of biomass precursors. Most importantly, our data suggest that the TCA cycle operates during methanol growth as both an oxidative and a reductive branch. This should be confirmed by further labeling experiments; however, simulations carried by forcing malate dehydrogenase (maldh) to operate in the opposite direction led to a worse fitting of the labeling data. On mannitol and arabitol, in contrast, the TCA cycle was used intensively, with absolute flux of 3.5 and 4.7 mmol/g (dry cell weight)/h (i.e., 0.59 and 1.32 C-mmol/C-mmol), respectively, for isocitrate dehydrogenase (idh) (Fig. 2B and C). These results agree with previous measurements of the actual usage of the TCA cycle during methylotrophic growth. At the transcript and protein levels, it has been suggested that the TCA cycle should be more active on mannitol than on methanol (21, 26). Activity assays in crude cell extracts also showed very low 2-oxoglutarate dehydrogenase (akgdh) activity on methanol (38). Finally, isotopic labeling experiments have shown slow isotopic enrichment of key TCA metabolites (citrate, 2-oxoglutarate, and fumarate) on methanol (27).

Although B. methanolicus also has glyoxylate shunt genes (BMMGA3_RS01750, 2.3.3.9; and BMMGA3_RS01755, 4.1.3.1), the reported expression levels suggest that this pathway does not carry a high flux under mannitol or under methanol growth conditions (21, 26). In agreement with these findings, the glyoxylate shunt fluxes estimated in this study were negligible under all the tested conditions (Fig. 2). Although the metabolic fluxes around the pyruvate node still require further characterization, the ^13^C-MFA supports activity of the anaplerotic reactions under all the tested conditions. In line with the noncircular operation of the TCA cycle, stronger activity of malic enzyme (mae) was observed on methanol than on mannitol and arabitol (i.e., relative flux of 0.66, 0.21, and 0.30 C-mmol/C-mmol, respectively) (Fig. 2B and C).

Overall, as reported before, the TCA cycle is mainly active under nonmethylotrophic conditions, and the glyoxylate shunt was inactive under our conditions. The novelty is the noncyclic operation of the TCA cycle under methylotrophic conditions.

### Analysis of cofactor usage with the various carbon sources.

To assess how B. methanolicus balances its redox and energy needs, we used the estimated fluxes from the ^13^C-MFA to infer production and consumption rates of ATP, NADH, and NADPH, as shown in Fig. 3. ^13^C-MFA is constrained by carbon balance and the distribution of the isotopic tracer, but unlike flux balance analysis, it is typically not constrained by cofactor balance. We used the estimated fluxes from the MFA and the expected stoichiometry of the associated reactions to assess the absolute rates of cofactor production and consumption. In the absence of specific measurements, we used the biomass requirements of Bacillus subtilis (8, 54). Additionally, since the samples were collected in metabolic pseudo-steady state, the production and consumption rates of each cofactor were assumed to be balanced; this allowed us to identify rates that would have been impossible to estimate otherwise such as the proportion of NAD (and ATP) formed from the respiratory chain (with a P/O = 1.5) and other production/consumption rates not accounted for by our other assumptions.

Despite their different growth rates, which influence their cofactor requirements for growth (Table 2), the cells grown on mannitol and arabitol had a similar absolute usage of NADH, NADPH, and ATP (Fig. 3). ATP and NADH came mostly from the same sources. However, the proportion of NADPH generated in the oxidative part of the PPP was three times higher under mannitol growth conditions than in arabitol (3.6 against 1.0 mmol/g [dry cell weight]/h), which is consistent with the higher fluxes observed in the oxidative part of the PPP on mannitol. This difference was compensated for by a higher flux in the TCA cycle and anaplerotic reactions for arabitol (7 against 5.3 mmol/g [dry cell weight]/h), in excess of the estimated NADPH requirements for growth and even contributing to the production of ∼5.6 mmol/g [dry cell weight]/h NADPH, whose consumption remains unaccounted for. Discrepancies between estimates of the cofactor requirements for biomass formation and those derived from measured isotopic data are typical for this kind of analysis (55, 56) and can be attributed to an underestimation of cofactor requirements, notably for nonessential processes such as cell motility (for ATP).

Comparing methylotrophic and nonmethylotrophic growth, it is not surprising that NADH was produced at a higher rate on methanol (39.2 mmol/g [dry cell weight]/h, versus 32.2 and 27.6 mmol/g [dry cell weight]/h on mannitol and arabitol, respectively) because of the conversion of methanol into formaldehyde by methanol dehydrogenase (57). This suggests that at the same growth rate (i.e., methanol versus mannitol), O_2_ consumption should be higher on methanol to provide the additional NAD^+^ required. The NADPH total production was estimated to be largely higher on methanol (14.3 mmol/g [dry cell weight]/h) than on mannitol and arabitol (Fig. 3). It was mainly provided by the oxidative part of the PPP (which would be part of the cyclic dissemination pathway; 4.6 mmol/g [dry cell weight]/h) as on mannitol but also by the linear formaldehyde detoxification pathways (4 mmol/g [dry cell weight]/h) and the anaplerotic reactions (i.e., malic enzyme; 5.1 mmol/g [dry cell weight]/h). These estimates suggest that both the linear detoxification pathways and malic enzyme play an important role in the generation of NADPH on methanol.

Overall, nonmethylotrophic growth on mannitol and on arabitol share the same features despite their associated different growth rates (with the notable exception of the contribution of the oxidative PPP for NADPH production). On methanol, however, we observed more differences in the origin of cofactor production, which clearly highlight a different metabolic state. Importantly, the linear detoxification pathways share with the PPP and the malic enzyme an important role in cofactor regeneration.

### Conclusion.

In summary, this MFA of B. methanolicus MGA3 provides three snapshots of its metabolic states for growth on methanol, mannitol, or arabitol. Isotopic data are consistent with prior knowledge of MGA3 methylotrophy, showing greater flux in the RuMP cycle than in the TCA cycle. The ^13^C-MFA provided new insights related to the utilization of cyclic RuMP versus linear dissimilation pathways and between the RuMP cycle variants; finally, the characterization of the arabitol assimilation pathway was completed using enzymatic data. In future studies, these validated flux maps will be used as references for constraint-based modeling to validate genome-scale model predictions. Overall, the information provided in this work and previous omics studies on B. methanolicus metabolism can be used to improve design strategies for new strains (i.e., by multi-omics analysis). For instance, the comparison between methanol and arabitol might help identify key control steps in the metabolic network that allows shifting from a cyclic (i.e., methanol with the Rump cycle) to a linear (i.e., arabitol with the PPP) mode of operation. The experimental path outlined here likely leads to B. methanolicus becoming a viable alternative to existing cell factories.

## MATERIALS AND METHODS

### Strain.

The B. methanolicus wild-type MGA3 (ATCC 53907) strain was used for metabolic flux analyses. Strains used for cloning and expression are described below in “Strains and culture conditions” and are listed in Table 3.

**TABLE 3:** Strains, primers, and plasmids used in this study

Strain, plasmid, or primer	Relevant characteristics or sequence (5′→3′)^*a*^	Reference, source, or characteristic
Strains		
E. coli DH5α	F^−^ *thi*-*1 endA1 hsdR17*(r^−^ m^−^) *supE44* Δ*lacU169* (φ80*lacZ*ΔM15) *recA1 gyrA96 relA1*	37
E. coli BL21 (DE3)	F^−^ *ompT hsdSB*(r_B_^−^ m_B_^−^) *gal dcm* (DE3)	70
B. methanolicus MGA3	Wild-type strain (ATCC 53907)	11
Plasmids		
pET16b	Amp^r^^*b*^, overproduction of decahistidine-tagged proteins in E. coli (pBR322 oriVE.c., *PT7*, *lacI*)	Novagen
pET16b-*atlD*	pET16b derivative for the production of B. methanolicus His_10_-tagged AtlD from E. coli BL21(DE3)	This study
pET16b-*atlF*	pET16b derivative for the production of B. methanolicus His_10_-tagged AtlF from E. coli BL21(DE3)	This study
Primers		
P192	agcttcctttcgggctttgttagcagccgTTAATCAATAGGTGTCAACAATAC	Amplification of *atlD* for pET16b-*atlD*
P193	gccatatcgaaggtcgtcatatgctcgagATGAAAGCTTTAGTCAAAAAAG	
P194	agcttcctttcgggctttgttagcagccgTTATGATTTTTCTGGATGGAAG	Amplification of *atlF* for pET16b-*atlF*
P195	gccatatcgaaggtcgtcatatgctcgagATGAAAGCATTAAAGCTGTATG	

a Overlapping regions are shown as lowercase letters.

b Amp^r^, ampicillin resistance.

### Nonstationary ^13^C fluxomics experiment.

**(i) Culture conditions and parameters.** For the carbon source methanol, two batch cultures were performed in 0.5-liter bioreactors (Multifors; INFORS HT, The Netherlands) with a working volume of 0.40 liters coupled to a Dycor ProLine Process mass spectrometer (AMETEK Process Instruments, USA). The culture medium per liter was 3.48 g Na_2_HPO_4_·12H_2_O, 0.606 g KH_2_PO_4_, 2.5 g NH_4_Cl, 0.048 g yeast extract, 1 ml of 1 M MgSO_4_ solution, 1 ml of trace salt solution, 1 ml of vitamin solution, 0.05 ml Antifoam 204, and 150 mM methanol. The trace salt solution per liter was 5.56 g FeSO_4_·7H_2_O, 0.027 g CuCl_2_·2H_2_O, 7.35 CaCl_2_·2H_2_O, 0.040 g CoCl_2_·6H_2_O, 9.90 g MnCl_2_·4H_2_O, 0.288 g ZnSO_4_·7H_2_O, and 0.031 g H_3_BO_3_. The vitamin solution per liter was 0.10 g d-biotin, 0.10 g thiamine-HCl, 0.10 g riboflavin, 0.10 g pyridoxine-HCl, 0.10 g pantothenate, 0.10 g nicotinamide, 0.02 g *p*-aminobenzoic acid, 0.01 g folic acid, 0.01 g vitamin B_12_, and 0.01 g lipoic acid. The precultures were grown in two 0.5-liter shake flasks containing 150 ml of the preculture medium and inoculated with cryostock of B. methanolicus wild-type MGA3 cells. The cultures were grown overnight at 50°C under shaking at 200 rpm and used to inoculate the reactors. The aeration rate of 1 vvm (volume of gas per volume of liquid per minute) was controlled by a mass flow meter (Multifors; INFORS HT, The Netherlands), and partial O_2_ pressure (pO_2_) was maintained at >25% throughout all cultures. Temperature, pH, and stirring speed were maintained at 50°C, pH 6.8 (with KOH 1 M), and 800 rpm, respectively. The N_2_, O_2_, Argon, CO_2_, ^13^C-CO_2_, and methanol concentrations in the bioreactors off-gas were measured on-line with the mass spectrometer.

To perform the pulse of tracer, 100 mM ^13^C-methanol (99% ^13^C; Euriso-Top, France) was added to the cultures at an optical density of 600 nm (OD_600_) of 2.5. Growth curves are available in Data Set S1 in the supplemental material.

**(ii) Quantification of cells and supernatant NMR analysis.** For determination of the dry weight of cells, a conversion factor of 0.389 g/liter (dry weight) of cells per OD_600_ unit was used. Supernatant samples were taken to analyze substrate methanol consumption as well as by-product formation by subtracting 1 ml of culture and centrifuging it at 13,000 × *g* for 60 s. Thereafter, supernatant was collected and stored until analysis at −20°C. Supernatant analysis was performed by 1D ^1^H NMR at 292°K, using a 30° pulse and a relaxation delay of 20 s, with an Avance 800 MHz spectrometer (Bruker, Germany). Deuterated trimethylsilyl propionate (TSP-d4) was used as an internal standard for quantification.

**(iii) Sampling and MS analysis of intra- and extracellular pool sizes.** When the cultures reached an OD_600_ of 2, metabolome samples were collected using the optimized method described in reference 28. Briefly, total broth quenching with correction for metabolites in the extracellular medium was performed in quadruplicates to assess the metabolite pool sizes in the two cultivations performed. Metabolites pool sizes were quantified (see Fig. S1) by ion chromatography tandem mass spectrometry (IC-MS/MS) using cell extract of Escherichia coli, cultivated on 99% [^13^C_6_]glucose (Euroisotop, France) as an internal standard (58). Liquid anion-exchange chromatography was performed as described previously (59).

10.1128/mSystems.00745-20.1 FIG S1 Measured intra- (A) and extracellular (B) pools of key metabolites of the central metabolism of B. methanolicus grown on methanol. Pools were measured by the “differential method” described in reference 10. Briefly, this method estimates the intracellular and extracellular metabolite pools from the quantification by ion chromatography mass spectrometry of the metabolites in the whole broth (intra- plus extracellular) and culture filtrates (extracellular). Samples were collected before the pulse of labeled methanol. The error bars represent the standard deviations from four technical replicates; the two biological replicates are represented by different colors. Metabolites measured were sedoheptulose 7-phosphate (S7P), phosphoenolpyruvate (PEP), 2 + 3 phosphoglycerate (2/3-PG), ribose 5-phosphate plus ribulose 5-phosphate plus xylulose 5-phosphate (R5P), ribose 1-phosphate (R1P), glucose 6-phosphate (G6P), fructose 1-phosphate (F1P), fructose 6-phosphate (F6P), mannose 6-phosphate (M6P), 6-phosphogluconate (6-PG), fructose 1,6-bisphosphate (FBP), adenosine monophosphate (AMP), adenosine diphosphate (ADP), adenosine triphosphate (ATP), UDP-glucose (UDP-Glc), shikimate 3P (Shi3P), fumarate (Fum), malate (Mal), orotate, citric acid (Cit), oxoglutarate (α-KG), succinate (Suc), trehalose 6-phosphate (Tre6P), aconitate (Aco), guanosine monophosphate (GMP), guanosine diphosphate (GDP), uridine diphosphate (UDP), cytidine diphosphate (CDP), uridine triphosphate (UTP), and cytidine triphosphate (CTP). Download FIG S1, TIF file, 1.4 MB. Copyright © 2020 Delépine et al. 2020 Delépine et al. This content is distributed under the terms of the Creative Commons Attribution 4.0 International license.

10.1128/mSystems.00745-20.2 FIG S2 Detection of formed arabitol phosphate in AtlD-catalyzed enzyme reactions using LC-MS. Chromatograms of detected pentitol phosphates (top) and their respective LC-MS spectra (bottom) of enzyme reactions using Xyl5P (A) and Ribu5P (B) as the substrates. The peaks for the given compounds were identified by characteristic mass spectra and retention time using standards for Xyl5P and Ribu5P or by comparison to previously reported *m/z* values for arabitol 5-phosphate (https://doi.org/10.1073/pnas.1423570112). Download FIG S2, TIF file, 0.1 MB. Copyright © 2020 Delépine et al. 2020 Delépine et al. This content is distributed under the terms of the Creative Commons Attribution 4.0 International license.

**(iv) Sampling and MS analysis of labeled metabolites.** Label enrichments in the intracellular metabolites were followed after performing a pulse of 100 mM ^13^C-methanol at an OD_600_ of 2.5. Whole broth (WB; internal plus external pools) and culture filtrate (CF; external pools) were sampled to indirectly track label incorporation in the intracellular metabolites. Specifically, 13 WB and 3 CF samples were collected in 3.5 min from each bioreactor. Exact sampling times can be seen in Data Set S1. IC-MS/MS quantification was used to analyze the isotopologues of each metabolite as described in reference 59. The metabolites analyzed were phosphoenolpyruvate (PEP), ribose 5-phosphate (Rib5P) plus Ribu5P plus Xyl5P, sedoheptulose 7-phosphate (Sed7P), 6-phosphogluconate (Gnt6P), glucose 6-phosphate (Glc6P), fructose 1,6-bisphosphate (FruBP), fructose 6-phosphate (Fru6P), glycerol 3-phosphate (Gly3P), 1,3-diphosphateglycerate (13PG), 3-phospho-d-glycerate (G3P) plus phosphoglycerate (PGA), citrate (Cit), aconitate (Aco), fumarate (Fum), malate (Mal), and succinate (Suc). After manual peak integration, the raw peak areas were corrected for the contribution of all naturally abundant isotopes using IsoCor software (60). Some cross-contamination was found in the isotopologues M + 4 of Aco and M + 2 and M + 3 of Gnt6P that were subsequently removed from the analysis (Data Set S1).

Additionally, the exact ratio between ^13^C-methanol and ^12^C-methanol after the pulse was measured by 1D ^1^H NMR as well as the evolution of ^12^CO_2_ and ^13^CO_2_ by the mass gas analyzer.

**(v) Mass balance.** Experimental data consistency of the measured rates was verified using standard data reconciliation procedures under the elemental mass balance constraints (61, 62). The biomass elemental composition used in the reconciliation procedure was taken from the closely related nonmethylotrophic bacterium Bacillus subtilis, CH_1.646_N_0.219_O_0.410_S_0.005_ (62, 63). The ash contents were considered to be 6% of the dry cell weight, average value obtained from different microorganisms (i.e., Escherichia coli, Aspergillus niger, Penicillium chrysogenum, and Klebsiella aerogenes [64]). After no proof of mismatch in the measurements, a better estimation of the physiological parameters was obtained as described in reference 61.

### Stationary ^13^C fluxomics experiments.

**(i) Culture conditions.** For the carbon sources mannitol and arabitol, the culture medium composition remained unchanged. The experiments were performed in 500-ml baffled shake flasks using 40 ml of medium, and cells were grown at 50°C and 200 rpm. Precultures contained yeast extract and 15 mM unlabeled mannitol or arabitol. They were inoculated as stated previously and grown overnight. The precultures were used to inoculate triplicate main cultures to an OD_600_ of 0.05 after centrifugation and resuspension in medium without yeast extract. The medium to perform the ^13^C MFA contained 15 mM [1-^13^C]mannitol, 15 mM [5-^13^C]arabitol, or 15 mM a mixture of 10% [1-^13^C]arabitol and 90% [2-^13^C]arabitol (99% ^13^C; Omicron Biochemicals, Inc., South Bend, IN, USA). An experimentally determined conversion factor of 0.22 g/liter (dry weight) of cells per OD_600_ unit was used. Growth curves are available in Data Set S1.

**(ii) Measurements of proteinogenic amino acids ^13^C-isotopologues.** Mannitol (or arabitol) cultures were sampled around 10 h (or 30 h) once they reached an OD_600_ of 1.3. The pellets obtained from the cellular extract were hydrolyzed 15 h at 110°C with 500 μl 6 N HCl. Samples were evaporated and washed twice with 500 μl of ultrapure water, evaporated to dryness, resuspended (625 μl, water), and diluted (1/1,000, water) for the mass spectrometry analysis.

Amino acids were separated on a pentafluorophenyl (PFP) column (150 mm, 2.1-mm inside diameter [i.d.], particle size, 5 μm; Supelco, Bellefonte, PA, USA). Solvent A was 0.1% formic acid in H_2_O and solvent B was 0.1% formic acid in acetonitrile at a flow rate of 250 μl/min. The gradient was adapted from the method used in reference 65. Solvent B was varied as follows: 0 min, 2%; 2 min, 2%; 10 min, 5%; 16 min, 35%; 20 min, 100%; and 24 min, 100%. The column was then equilibrated for 6 min under the initial conditions before the next sample was analyzed. The volume of injection was 20 μl.

High-resolution experiments were performed with an Ultimate 3000 high-performance liquid chromatography (HPLC) system (Dionex, CA, USA) coupled to an LTQ Orbitrap Velos mass spectrometer (Thermo Fisher Scientific, Waltham, MA, USA) equipped with a heated electrospray ionization probe. MS analyses were performed in positive Fourier transform MS (FTMS) mode at a resolution of 60,000 (at 400 *m/z*) in full-scan mode, with the following source parameters: capillary temperature, 275°C; source heater temperature, 250°C; sheath gas flow rate, 45 AU (arbitrary unit); auxiliary gas flow rate, 20 AU; S-lens radio frequency (RF) level, 40%; source voltage, 5 kV. Isotopic clusters were determined by extracting the exact mass of all isotopologues, with a tolerance of 5 ppm. Experimental carbon isotopologue distributions (CIDs) of alanine, glycine, valine, serine, threonine, phenylalanine, aspartate, glutamate, histidine, isoleucine, leucine, lysine, arginine, tyrosine, proline, and methionine were obtained after correction of raw MS data for naturally occurring isotopes other than carbon, using IsoCor (60). Careful inspection of the CIDs revealed an overall excellent reproducibility between both the technical and biological replicates (Data Set S1). However, M + 0 of valine and M + 0 to M + 1 of glycine had higher variability, which could be due to the signals being closer to the noise level.

**(iii) NMR measurements.** Concentrations in supernatants were measured by 1D ^1^H NMR at 290°K using a 30° angle pulse, and a presaturation of water signal was applied during a relaxation delay of 8 s. TSP-d4 was used as an internal standard for calibration and quantification.

The measurement of isotopomers and specific enrichments of targeted biomass components were performed using the same samples used for proteinogenic amino acids and ^13^C-isotopologues MS analysis, which were redried and suspended in 200 μl D_2_O (0.1% DCl). The positional isotopomer distribution of alanine C-2 and C-3 was extracted from the analysis of ^13^C-^13^C couplings in two-dimensional (2D) ^1^H-^13^C heteronuclear single quantum coherence (HSQC) experiments (66). The carbon isotopic enrichments of alanine (C-2 and C-3) and histidine (C-2 and C-5) were extracted from the analysis of ^1^H-^13^C couplings using 2D zero quantum filtered-total correlation spectroscopy (ZQF-TOCSY) (67).

NMR spectra were collected on an Avance III 800 MHz spectrometer (Bruker, Germany) equipped with a 5-mm z-gradient QPCI cryoprobe. Every acquisition, 1D, 2D, and absolute quantifications were performed on 1D ^1^H spectra using TopSpin 3.5 (Bruker, Germany).

### Models and simulations.

**(i) Metabolic flux analysis software.** All simulations needed for metabolic flux analysis (MFA) on methanol, mannitol, and arabitol were performed with influx_si software v4.4.4 (68), either in stationary or nonstationary mode. “influx” has the advantages of allowing for the integration of labeling data coming from different experimental setups (MS and NMR) and supporting several integration strategies (stationary, nonstationary, and parallel labeling).

All input and output files needed for reproducibility are available in an archive in https://fairdomhub.org/data_files/3269?version=2. Importantly, this includes the models in FTBL and SBML file formats.

**(ii) Metabolic models.** We designed the models to cover central carbon metabolism and biomass needs of B. methanolicus. influx_si uses a nonstandard legacy file format (FTBL) to encode the metabolic network and the associated atom-atom transitions. This format centralizes the metabolic network with all biological measurements, i.e., metabolite pool sizes, fluxes, and carbon isotopologue distribution. Consequently, we developed distinct model files for each carbon source. Models share the same nomenclature and the same general topology between them, which is displayed in Fig. 2 and detailed in Data Set S1.

Assimilation pathways of methanol, mannitol, and arabitol were included when relevant to explain the incorporation of the tracer. The CO_2_ pool was explicitly modeled within the system to allow for reincorporation of tracer via CO_2_. Amino acid synthesis pathways were modeled as part of biomass needs along with important precursors. The biomass equation was borrowed from a Bacillus subtilis genome-scale model (69). The reaction for phosphoenolpyruvate carboxykinase (BMMGA3_RS13120) was not included in the final model, as the associated expression level was rather low in proteomics (26) and transcriptomics (21, 25) studies.

Unless otherwise stated, each culture replicate was processed independently to estimate fluxes for their respective carbon source condition.

For MFA on mannitol, we exploited carbon isotopologue distribution data of proteinogenic alanine, glycine, valine, serine, threonine, phenylalanine, aspartate, glutamate, histidine, isoleucine, leucine, lysine, arginine, tyrosine, proline, and methionine. Acetate production was modeled with an export flux from acetyl-CoA, to which we associated the acetate flux measured from supernatant data. Acetyl-CoA was further constrained using acetate positional labeling measured by 1D ^1^H NMR spectroscopy from the supernatant.

For MFA on arabitol, we averaged the proteinogenic labeling measurements of the [5-^13^C]arabitol experiment and exploited it as a parallel labeling data set for each biological replicate of the [1/2-^13^C]arabitol experiment. We analyzed the same proteinogenic amino acids as those mentioned above for mannitol. No acetate was observed in the supernatant, and the associated export reaction was consequently excluded from this model. Additionally, we exploited specific labeling enrichment of histidine, alanine, and ribulose-5-P and positional isotopomer data of alanine from labeling samples as described in “NMR measurements.”

For MFA on methanol, the nonstationary nature of the experiment and the subsequent importance of the pools on flux distributions forced us to explicitly examine most of the reactions of the central metabolism that were lumped for the stationary models. Measurements described above were used to constrain intra- and extracellular pools (see “Sampling and MS analysis of intra- and extracellular pool sizes”) and isotopologue profiles (see “Sampling and MS analysis of labeled metabolites”) through the influx_si optimization process. Only the measurement of the CO_2_ from the exhaust gas was used but not its labeling dynamic. The exchange fluxes of CO_2_ and methanol (feed and evaporation) were also exploited. No acetate production was observed. Because some isotopologues of Gnt6P (M + 2 and M + 3) could not be quantified, these isotopic measurements could not be included in the influx_si model; hence, the flux through the oxidative PPP could not be accurately resolved. To exploit these partial data and estimate the flux through the oxidative PPP, we applied the ScalaFlux approach (37), which allows us to quantify fluxes through a given metabolic subnetwork of interest by modeling label propagation directly from the metabolic precursor of this subnetwork. The flux calculations are thus purely based on information from within the subnetwork of interest, and no additional knowledge about the surrounding network is required. Briefly, we defined a metabolic subnetwork containing two reactions: (i) one reaction converting Glc6P into Gnt6P, and (ii) one sink reaction operating at the same rate to balance the Gnt6P pool, consistent with the metabolic steady-state assumption. This metabolic model assumes direct formation of Gnt6P from Glc6P. Flux calculations were based on the time course profile of M + 0 (relative to M + 0, M + 1, M + 4, M + 5, and M + 6), which showed the highest range of variation during this experiment and was thus less affected by measurement noise. Fraction M + 0 of Glc6P was corrected for the presence of an extracellular unlabeled pool by normalizing its profile to that for the isotopic steady-state of Gnt6P. Experimental labeling dynamics of the precursor of this subnetwork (Glc6P) were used as label input, and the flux through the oxidative PPP was estimated by fitting the concentration and M + 0 profile of Gnt6P. The goodness of fit was evaluated using a chi-square test, and the flux precision was estimated using the Monte-Carlo approach (with 200 iterations).

**(iii) Quality checks and flux conversion.** Experimental data were fitted to our models as described above. For each culture replicate, we performed a Monte Carlo sensitivity analysis (*n* = 100) on the fit to assess its robustness to small variations around the fitted values. We also performed a chi-squared goodness-of-fit statistical test to ensure that simulated data for each biological replicate were significantly close to experimental data. All tests were significant with a significance level (α) of 0.05 (https://fairdomhub.org/data_files/3269?version=2). For convenience, we provide figures of measured versus simulated data points (see Fig. S3).

10.1128/mSystems.00745-20.3 FIG S3 Measured versus estimated labeling proportion under methanol (A), mannitol (B), and arabitol (C) conditions. Each data point refers to the proportion of one of the isotopologues from the carbon isotopologue distribution (CID) of a metabolite used to constrain the models, i.e., either metabolites from central metabolism (A) or proteinogenic amino acids (B and C) as described in Materials and Methods. On the *y* axis, the measured value by mass spectrometry; on the *x* axis, the value was simulated and used by influx after the optimization process to estimate the fluxes. There is a strong linear correlation between simulated and measured values, which is a good visual indicator of the coherence of the model with experimental data. Download FIG S3, TIF file, 2.4 MB. Copyright © 2020 Delépine et al. 2020 Delépine et al. This content is distributed under the terms of the Creative Commons Attribution 4.0 International license.

On mannitol and arabitol, the precision of the fluxes through malate dehydrogenase (maldh; BMMGA3_RS12590, 1.1.1.37), malic enzyme (mae; BMMGA3_RS12650, 1.1.1.38 or 1.1.1.40), and pyruvate carboxylase (pyc; BMMGA3_RS05255, 6.4.1.1) was not satisfactory. We thus applied a flux minimization analysis (70) included in the influx_si software (i.e., option sln) to estimate the minimal flux (i.e., lower bound) values that could be carried through those 3 reactions.

Since both the substrate uptake rates (see Table 2) and the content of carbon of the substrates (from 1 to 6 carbon atoms) differed widely, the estimated fluxes were normalized as follows. All the estimated fluxes (in millimoles per gram [dry cell weight] per hour) were multiplied by the number of carbon atoms of its corresponding reactants (Data Set S1). The resulting metabolic fluxes (in millimoles of carbon per gram [dry cell weight] per hour) were then divided by the substrate uptake rate expressed in millimoles of carbon per gram [dry cell weight] per hour. The resulting normalized fluxes expressed in millimoles of carbon per millimole of carbon from the carbon source (given in Data Set S1) were used to allow a direct comparison across the different conditions.

### Analysis of arabitol phosphate dehydrogenases AtlD and AtlF.

**(i) Strains and culture conditions.** In this study, Escherichia coli DH5α (71) was used as the standard cloning host, and recombinant protein production was carried out with E. coli BL21(DE3) (72). A summary of the strains, primers, and plasmids constructed and used in this study can be found in Table 3. E. coli strains were routinely cultivated at 37°C and 180 rpm in lysogeny broth (LB) or on LB agar plates supplemented with 100 μg ml^−1^ ampicillin and 0.5 mM isopropyl-β-d-thiogalactopyranoside (IPTG) when relevant.

**(ii) Recombinant DNA work.** Molecular cloning was performed as previously described (73) using primer sequences listed in Table 3. Total DNA isolation from B. methanolicus was performed as described in reference 74. Inserts were amplified by PCRs with ALLin HiFi DNA polymerase (HighQu, Kraichtal, Germany) and purified with the NucleoSpin Gel and PCR Clean-up kit (Macherey-Nagel, Düren, Germany). Plasmids were constructed from PCR-generated fragments and pET16b vector cut with restriction enzymes using the isothermal DNA assembly method (75). The GeneJET Plasmid Miniprep kit (Thermo Fisher Scientific, Waltham, MA, USA) was used for plasmid isolation. For the transformation of chemically competent E. coli cells, the procedure described in reference 76 was used. Colony PCRs were performed using *Taq* polymerase (New England Biolabs, Ipswich, MA) with primers P192, P193, P194, and P195 (Table 3). All cloned DNA fragments were verified by sequencing (Sequencing Core Facility, Bielefeld University).

**(iii) Overproduction and purification of AtlD and AtlF.** Plasmids for protein production using E. coli BL21(DE3) were constructed on the basis of pET16b (Novagen, Madison, WI, USA) and are presented in Table 3. The *atlD* or *atlF* gene was PCR amplified from B. methanolicus MGA3 genomic DNA using the primers P192 and P193 or P194 and P195, respectively (Table 3). The resulting product was joined with BamHI-digested pET16b by applying the isothermal DNA assembly method (75), resulting in pET16b-*atlD* or pET16b-*atlF*. The pET16 vector allows for production of N-terminal His_10_-tagged proteins. Protein production and purification were performed according to the indications in reference 77, except for cell lysis, which was performed by sonication (UP 200 S; Dr. Hielscher GmbH, Teltow, Germany) on ice at an amplitude of 55% and a duty cycle of 0.5 for 8 min with a pause in-between. Supernatants were subsequently filtered using a 0.2-μm filter and purified by nickel affinity chromatography with nickel-activated nitrilotriacetic acid-agarose (Ni-NTA) (Novagen, San Diego, CA, USA). His-tagged AtlD and AtlF proteins eluted with 20 mM Tris, 300 mM NaCl, 5% (vol/vol) glycerol, and 50, 100, 200, or 400 mM imidazole and were analyzed by 12% SDS-PAGE (78). Fractions showing the highest protein concentrations (with 100 and 200 mM or 100, 200, and 400 mM imidazole for AtlD and AtlF, respectively) were pooled, and protein concentrations were measured according to the Bradford method (79) using bovine serum albumin as the reference. The purified protein was subsequently applied for enzymatic assays.

**(iv) Arabitol phosphate dehydrogenase enzymatic assays.** Determination of purified AtlD and AtlF activities in the reductive reaction using Xyl5P or Ribu5P as the substrate were performed as previously described (23). The assay mixture contained 20 mM Tris-HCl buffer (pH 7.2), 1 mM dithiothreitol (DTT), 0.04 to 0.3 mM NADH or NADPH, 0.03 to 0.6 mM Xyl5P or 0.2 to 4 mM Ribu5P, and 0.01 to 0.04 mg AtlD or 0.2 to 0.4 mg AtlF in a total volume of 1 ml. The oxidation rate of NADH or NADPH was monitored at 340 nm and 30°C for 3 min using a Shimadzu UV-1202 spectrophotometer (Shimadzu, Duisburg, Germany). To confirm the presence of arabitol phosphate in the enzyme reaction mixtures after reduction of Xyl5P and Ribu5P, samples were subjected to liquid chromatography-mass spectrometry (LC-MS) analyses according to the procedure described in reference 50.

### Data availability.

Gene loci mentioned throughout the text are from NCBI annotation of B. methanolicus MGA3 genome NZ_CP007739.1. Data Set S1 and supplementary figures and text are available in the online version of this paper. Raw models and input and output files for influx software can be downloaded from https://fairdomhub.org/data_files/3269?version=2.
