# Supplementary figures and images for: Charting the Metabolic Landscape of the Facultative Methylotroph Bacillus methanolicus
Source: mSystems. 2020 Sep 22;5(5):e00745-20. doi: 10.1128/mSystems.00745-20 (PMC7511216; doi:10.1128/mSystems.00745-20)

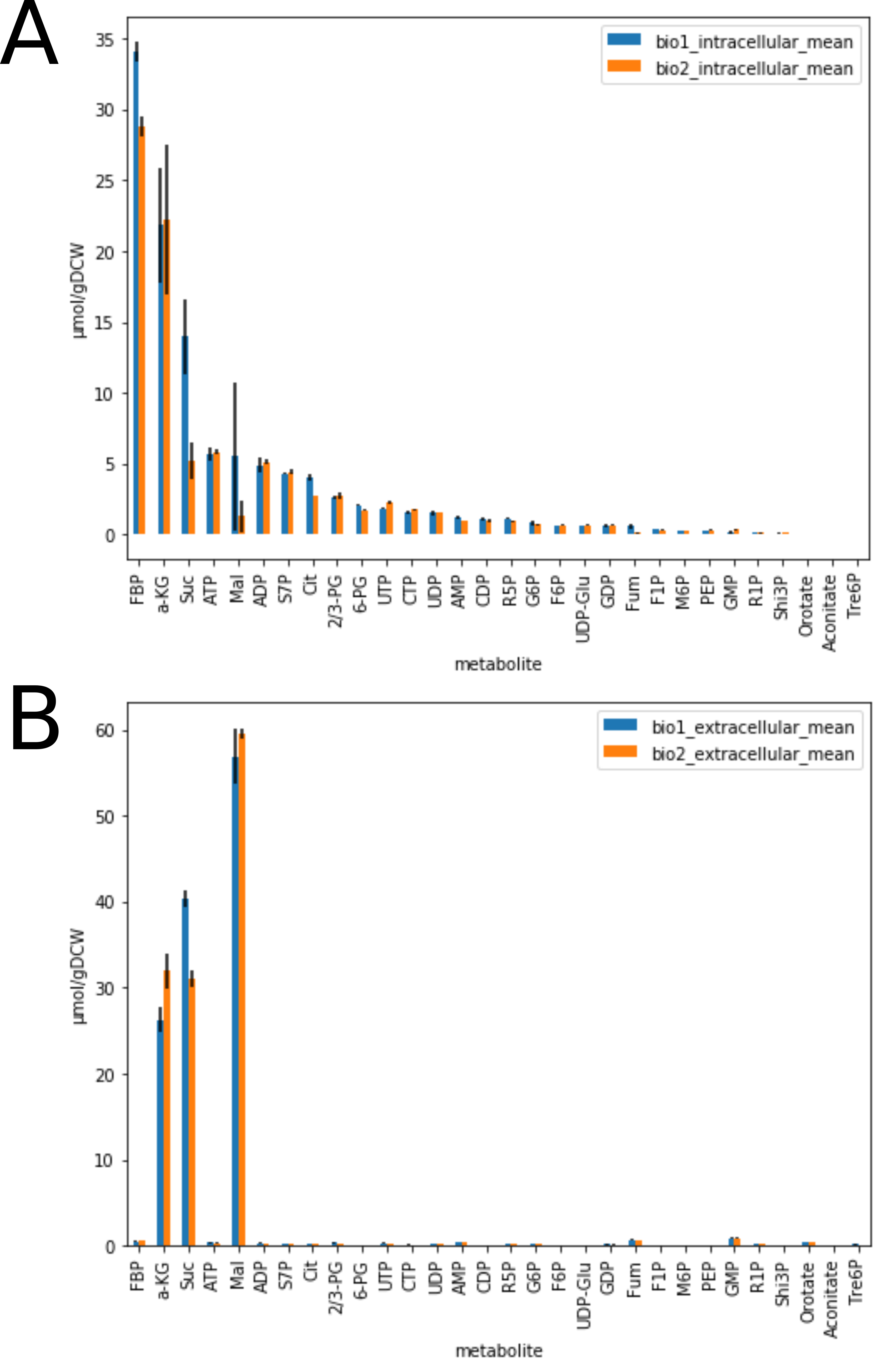

Supplement: FIG S1 [file mSystems.00745-20-sf001.tif]

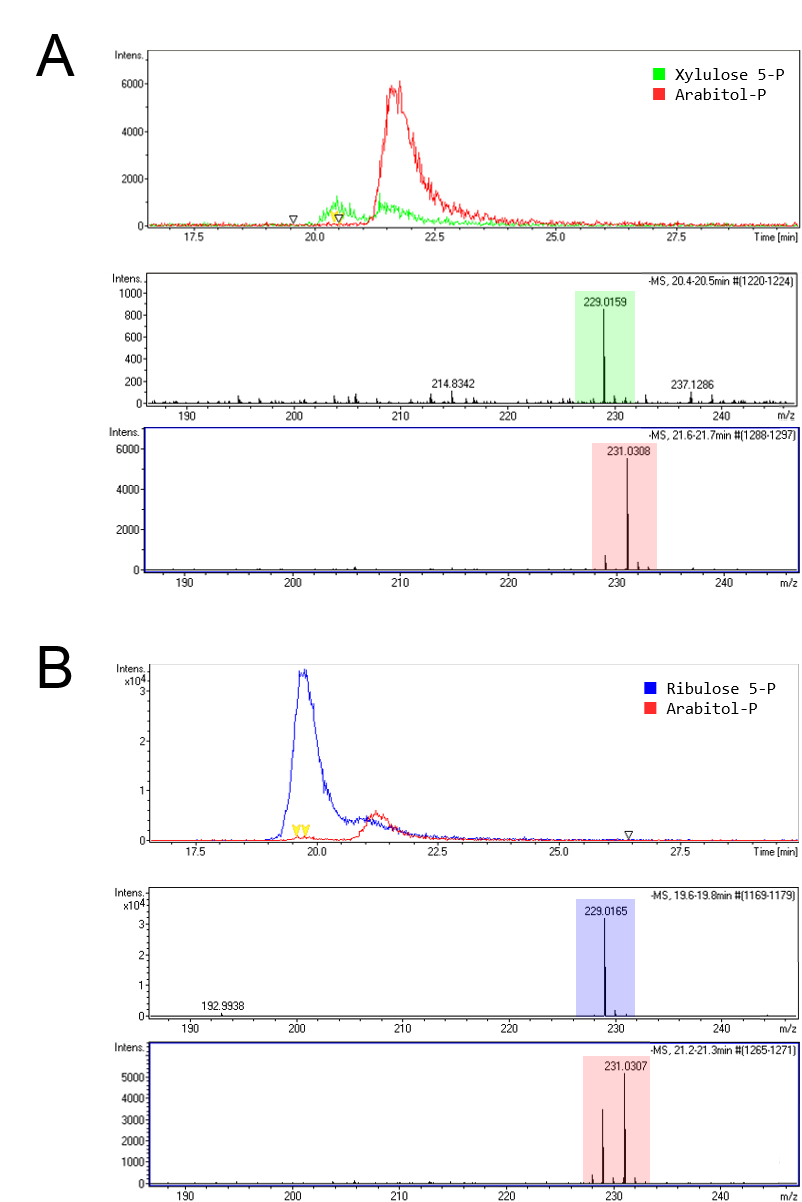

Supplement: FIG S2 [file mSystems.00745-20-sf002.tif]

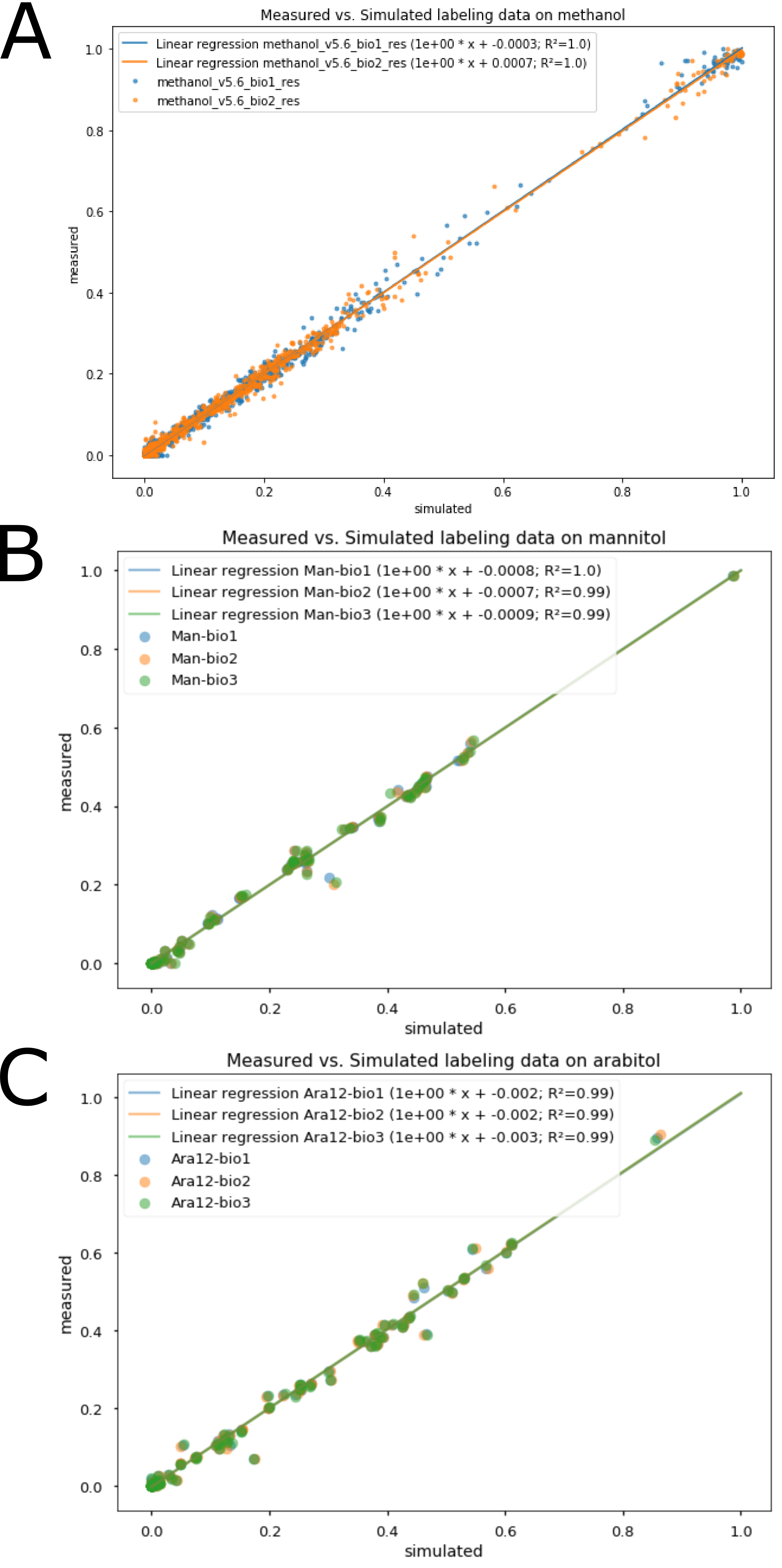

Supplement: FIG S3 [file mSystems.00745-20-sf003.tif]
